# p53 regulates the mevalonate pathway in human glioblastoma multiforme

**DOI:** 10.1038/cddis.2015.279

**Published:** 2015-10-15

**Authors:** C Laezza, A D'Alessandro, L Di Croce, P Picardi, E Ciaglia, S Pisanti, A M Malfitano, M Comegna, R Faraonio, P Gazzerro, M Bifulco

**Affiliations:** 1 Institute of Endocrinology and Experimental Oncology, IEOS CNR, Naples, Italy; 2 Department of Medicine and Surgery, University of Salerno, Baronissi (SA), Italy; 3 Department of Pharmacy, University of Salerno, Via Giovanni Paolo II, 132, Fisciano, Italy; 4 Centre de Regulacio Genomica (CRG), Universitat Pompeu Fabra, Dr. Aiguader 88, Barcelona, Spain; 5 Department Molecular Medicine and Medical Biotechnologies, University of Naples ‘Federico II' via S. Pansini, 5, Naples, Italy; 6 CEINGE, Biotecnologie Avanzate, Naples, Italy

## Abstract

The mevalonate (MVA) pathway is an important metabolic pathway implicated in multiple aspects of tumorigenesis. In this study, we provided evidence that p53 induces the expression of a group of enzymes of the MVA pathway including 3′-hydroxy-3′-methylglutaryl-coenzyme A reductase, MVA kinase, farnesyl diphosphate synthase and farnesyl diphosphate farnesyl transferase 1, in the human glioblastoma multiforme cell line, U343 cells, and in normal human astrocytes, NHAs. Genetic and pharmacologic perturbation of p53 directly influences the expression of these genes. Furthermore, p53 is recruited to the gene promoters in designated p53-responsive elements, thereby increasing their transcription. Such effect was abolished by site-directed mutagenesis in the p53-responsive element of promoter of the genes. These findings highlight another aspect of p53 functions unrelated to tumor suppression and suggest p53 as a novel regulator of the MVA pathway providing insight into the role of this pathway in cancer progression.

The control of cellular metabolism is essential for a normal cell behavior, and the role that aberrant cellular metabolism has in cancer is becoming increasingly evident. During the course of tumorigenesis, changes in metabolism enable cells to survive and proliferate under adverse conditions that would directly arrest or kill a normal cell.^1^ Recent work has highlighted increased mevalonate (MVA) synthesis in malignant cells as consequence of increased levels and catalytic efficiency of 3′-hydroxy-3′-methylglutaryl-CoA reductase (HMGCR), the rate-limiting enzyme of cholesterol (CHO) biosynthesis that catalyzes the formation of MVA ^2^ (Scheme 1). In addition, MVA is required for a number of cellular processes including DNA synthesis and proliferation.^3^ It is also the precursor of non-sterol isoprenoids endowed with a variety of functions including prenylation of growth-regulating proteins and oncoproteins.^4^ Elevated MVA synthesis has been reported in malignant breast cancer,^5^ lung,^6^ leukemia and lymphoma^7^ and hepatoma.^8^ Most recent results have shown that the administration of exogenous MVA to xenograft-bearing mice promoted tumor growth.^9^ Taken together, these results suggest that MVA pathway may have an important role in human malignancies. Indeed, recent transcriptional profiling demonstrated that CHO and lipid metabolism are linked to cellular transformation.^10^ However, it is not known whether the alterations of MVA pathway contribute to cancer etiology or whether its dysregulation occurs as a consequence of transformation. Given the importance of metabolic reprogramming in tumor development, it is not surprising that many oncogenes and tumor-suppressor genes have been shown to fine-tune these pathways.^1, 11^ Their roles in carcinogenesis have traditionally been attributed to the ability to regulate the life and/or cell death.^12^ Anyway, evidence for an alternative concept, which the primary functions of activated oncogenes and inactivated tumor suppressors are to reprogram cellular metabolism, has continued to build over the past years. Among them, p53 seems to have an important role in regulating several aspects of cellular metabolism that are now being explored not only in cancer but in other diseases and in development.^13^ The p53 tumor-suppressor protein is a key factor in the cellular defense against carcinogenesis. It is a transcription factor that responds to numerous extrinsic and intrinsic challenges to the cell, including DNA damage, oncogene activation and hypoxia, to promote a variety of responses depending on the type, severity and persistence of the stress.^13^ An emerging striking observation is that by transcriptional activation and other means, p53 is able to contribute to the regulation of several pathways as oxidative phosphorylation, glutaminolysis, insulin sensitivity, nucleotide biosynthesis, mitochondrial integrity, fatty acid oxidation, antioxidant response, autophagy and mTOR signaling.^14^ These evidences underscore the importance of the gene regulation achieved by p53. In this study, we found that p53 regulates the expression levels of some enzymes of the MVA metabolism in a human glioblastoma multiforme cell line and in their normal astrocytic counterpart. It recruited the genes promoters of these enzymes to activate their transcription. These results reveal a novel role of p53 in the systemic regulation of isoprenoid homeostasis.

## Results

### p53 induces the expression of MVA metabolism-related genes in human glioblastoma cells

**Figure 1:**
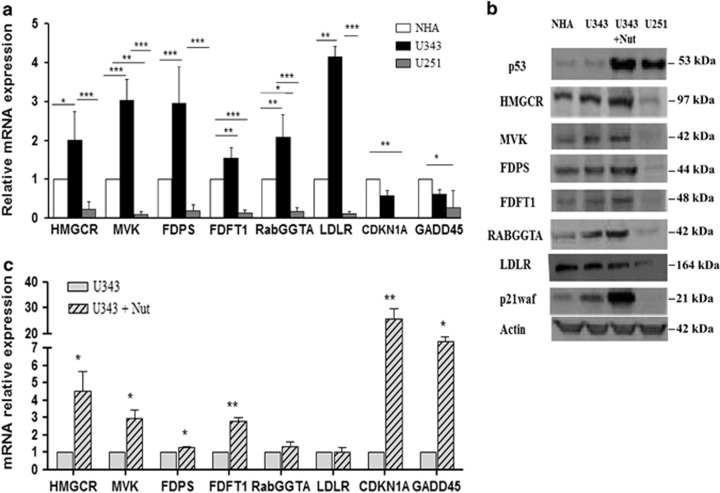
HMGR, MVK, FDPS, FDFT1, RabGGTA and LDLR expression in different cell lines. (**a**) qRT-PCR analyses in U343 cells with wild-type p53, U251 cells bearing a mutant p53 (R273H) and NHA cells used as reference cell line for basal levels. CDKN1A and GADD45 genes served as a positive control. All the data represented mean±S.D. of five independent experiments. Statistical analysis was conducted with one-way ANOVA and columns indicated with asterisks represent a significant difference in gene expression levels (***P≤0.001; **P≤0.01; *P≤0.05) compared with NHA control cells. (**b**) Western blot analyses of protein levels of the MVA pathway enzymes and p21waf. A representative actin was used for all samples as control. (**c**) qRT-PCR analysis of MVA pathway enzymes was conducted for U343 cells treated or not with Nut at 10 *μ*M for 24 h. Data are representative of three different experiments. A two-tailed *t*-test was conducted as statistical analysis. Columns indicated with asterisks represent significant increase compared with unmarked columns (**P≤ 0.01; *P≤0.05)

**Figure 2:**
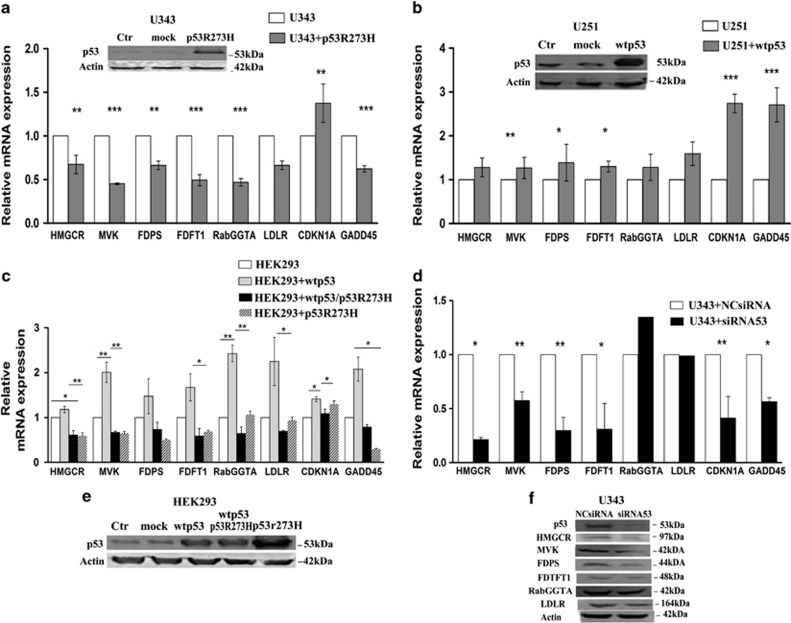
Analysis of mRNA levels of enzymes related to MVA pathway upon genetic perturbation of p53. qRT-PCR analysis of MVA pathway enzymes in (**a**) U343 cells untransfected and transfected with empty vector control (mock) or with mutant (R273H) p53. Data were analyzed with a two-tailed *t*-test. Western blot showed p53 protein levels following transfections with the plasmid constructs in U343 cells (ctr=untransfected cells; mock=cells transfected with empty vector; U343+p53(R273H)=cells transfected with mutant p53). (**b**) U251 cells transfected with empty vector control or with vector containing wild-type p53 (pCMVp53). Western blot shows p53 protein levels following transfections. (**c**) HEK293 cells transfected with control empty vector (mock), or with pCMVp53 alone, co-transfected with construct containing with mutant p53 or transfected with (R273H) p53 alone. Western blot shows the transfection efficiency. Data are mean±S.D. of three independent experiments. One-way ANOVA analysis indicated a significant difference in genes expression (**P≤0.001; *P≤0.05). (**d**) Knockdown of wt p53 from U343 cell line: cells transfected with siRNA p53 downregulated the expression and the protein levels of enzymes of MVA pathway (**f**). A representative actin was used for all samples as loading control. (**e**) Western blot analyses shows the overexpression of p53 after transfection in HEK293 cell line in the several experimental conditions. ***P≤0.001

High or deregulated activity of the MVA pathway has been demonstrated in several different tumors.^15, 16^ In order to study the activity of this pathway in glioblastoma multiforme, we compared gene expression profiles of six genes relative to MVA pathway, including HMGCR, MVA kinase (MVK), farnesyl diphosphate synthase (FDPS), farnesyl diphosphate farnesyl transferase 1 (FDFT1), Rab geranylgeranyl transferase 1*α* (RabGGTA) and LDL receptor (LDLR) (Scheme 1) in U343 cells expressing wild-type p53 and U251 cells with mutant p53 (R273H), carrying a mutation abolishing the DNA-binding activity.^17^ Normal human astrocytes, NHAs, were used in comparison. Quantitative real-time PCR (qRT-PCR) measurements were conducted for mRNA levels of aforementioned genes (Supplementary Table 1). As shown in Figure 1a, the obtained data revealed that they were significantly upregulated at basal level in U343 cells expressing wild-type p53 and downregulated in U251 cells with mutant p53 (R273H), compared with NHA, a NHA cell line. In this context, the mRNA levels of CDKN1A (p21waf) and GADD45, known p53 target genes,^18^ paralleled with p53 activity. In order to verify if p53 activation induced the expression of the genes involved in the MVA pathway, we performed qRT-PCR measurement of the mentioned genes in U343 cells treated with Nutlin 3a (Nut), which is known to stabilize and activate p53.^19^ As shown in Figure 1c, we observed that the expression levels of MVA pathway genes were induced in Nutlin 3a-treated cells compared with untreated cells. Furthermore, we evaluated by western blot analysis (Figure 1b) the protein levels of the enzymes of the MVA pathway and observed that they were overexpressed in NHA cells and U343 cells untreated and treated with Nutlin 3a in comparison with U251 cells, thus confirming proper p53 activity in our experimental setup (Figure 1c). In parallel, in order to demonstrate the transcriptional regulation of these genes by p53, we transfected U343 cells with either an empty vector (mock) or a vector encoding the mutant p53 (R273H) (Figure 2a). The ectopic expression of a dominant-negative mutant p53 (R273H) in U343 cells significantly attenuated the mRNA levels of the six MVA pathway genes analyzed (Figure 2a). The transfection efficiency has been confirmed by western blot analysis (Figure 2a). To support the role of p53 in transcriptional regulation of the enzymes of MVA pathway, we knocked down p53 using a specific p53-siRNA. We observed that the reduction of p53, as shown in western blot in Figure 2f, turned down both the expression and protein levels of the tested enzymes (Figures 2d and f). Then, to corroborate our findings, we examined the expression of all these genes following ectopic expression of wild-type p53 in HEK293 cells. As shown in Figure 2c, the overexpression of wild-type p53 in HEK293 cells, significantly induced the mRNA levels of all genes compared with cells transfected with empty vector (mock), instead the overexpression of mutant p53 alone reduced the expression of analyzed genes. The western blot in Figure 2e shows the transfection efficiency. The role of p53 on these genes was confirmed, at least in part, in U251 cells, after transfection with a vector bearing wild-type p53 (Figure 2b). In order to further generalize our findings, we tested the ability of p53 to activate the MVA metabolism-related genes in human adenocarcinoma breast cancer cell lines MCF7 with wild-type p53 and MDA MB 231 with p53 mutant (R280T)^17^ and in human adenocarcinoma colon cancer cell lines HCT116 with wild-type p53 and SW620 with p53 mutant (R273H).^17^ In accordance with human glioblastoma cells used in this study, levels of six genes were increased in cell lines with wild-type p53 compared with cell lines with mutant p53 (Supplementary Data,Supplementary Figure S1).

### p53 is recruited to p53-responsive elements in the promoters of MVA pathway-related genes thereby inducing their transcription

**Figure 3:**
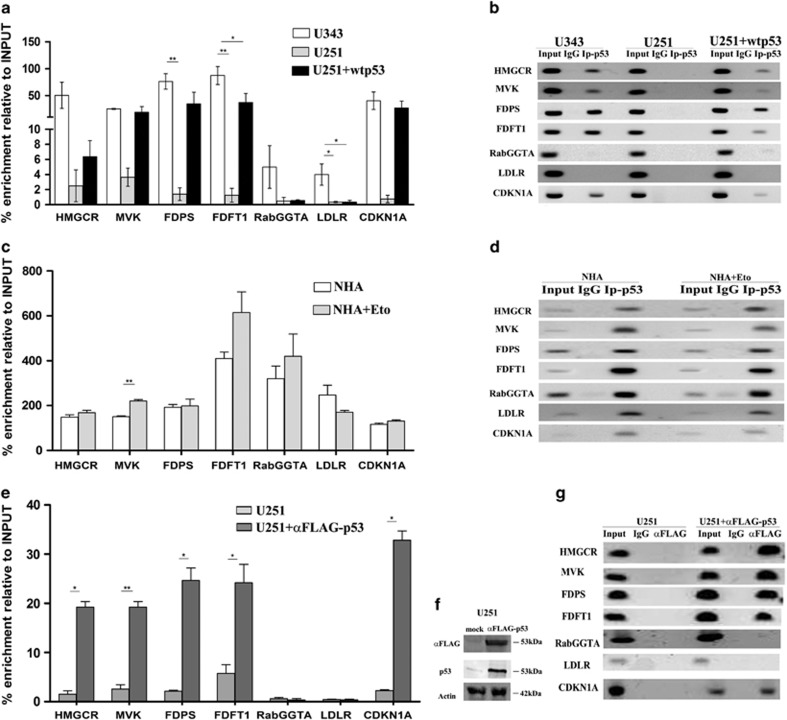
ChIP assays in glioblastoma cells. Chromatin samples from (**a**) U343 cells, U251 cells and pCMVp53 transfected U251 cells were immunoprecipitated in the presence of control rabbit IgG or with specific antibody anti-p53. p53 is immunoprecipitated along with p53REs of promoter of HMGR, MVK, FDPS, FDFT1. qRT-PCR analysis was conducted using primers set specific for putative p53REs (Supplementary table 3). Obtained results were normalized to 1% input. (**b**) Representative agarose gels of PCR products of ChIP assay using an antibody against p53 and anti-rabbit IgG as control of U343 cells, U251 cells and U251 transfected with p53wt. (**c**) qRT-PCR analysis of p53REs co-immunoprecipitated with p53 in NHA cells untreated and treated with eto at 25 *μ*M for 24 h. All the data are reported as mean ±S.D. of two independent experiments. One-way ANOVA was performed to indicate significant differences in genes expression. CDKN1A promoter was used as a positive control. (**d**) Representative agarose gels of PCR products of ChIP assay using an antibody against p53 and anti-rabbit IgG as control of NHA and NHA treated with eto. (**e**) Chromatin samples from U251 transfected with FLAG-p53 or with an equivalent amount of empty vector were immunoprecipitated with anti-mouse IgG, anti-FLAG or p53 antibodies as indicated. qRT-PCR analysis was conducted using primers set specific for putative p53REs. (**f**) Western blot reports the amounts of FLAG-p53 and of p53 in one typical experiment. Actin was made as control of loading. (**g**) Representative agarose of PCR products of ChIP assay using an antibody against FLAG and anti-rabbit IgG as control of U251 cells transfected with FLAG-p53 and equivalent amount of empty vector. One-way ANOVA analysis indicated a significant difference in genes expression (*P≤0.05; **P≤0.001)

**Figure 4:**
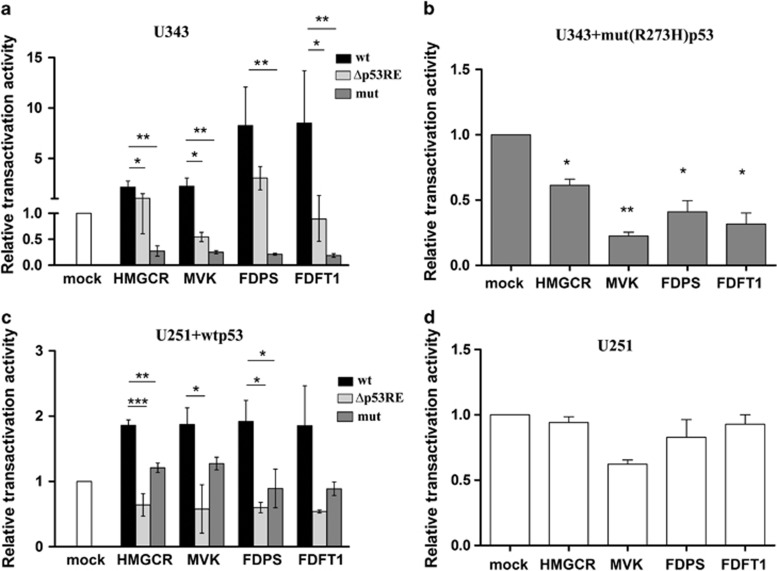
Luciferase reporter assays in glioblastoma cells. (**a**) U343 cells were transiently transfected with three different pGL3 plasmids wt: containing the promoter regions containing p53RE (wild type) from HMGCR, MVK, FDPS and FDFT1 gene sequences, Δp53RE: the constructs with deleted-cloned sequences of the mentioned genes (Δp53RE) and mut: the constructs containing promoter regions with single mutation of p53 of HMGCR, MVK, FDPS and FDFT1 genes (mut). (**b**) U343 cells were co-transfected with mut (R273H) p53 plasmid and with constructs containing promoter regions. (**c**) wt p53-transfected U251 cells were co-transfected with constructs containing wt and mutated promoter regions of enzymes of MVA pathway. (**d**) U251 cells were transfected with promoter regions–constructs of MVA enzymes. Results were normalized for *β*-galactosidase activity and compared with results obtained in cells transfected with empty vector (mock). Data represent mean±S.D. of three independent experiments and statistical analysis was performed with one-way ANOVA, *P≤0.05; **P≤0.01; ***P≤0.001

p53 induces transcription of its primary target genes by a direct binding of PuPuPuC(AT)(TA)GPyPyPy sequence presented in p53-responsive element (p53RE) of DNA regulatory elements (promoters and enhancers).^20^ Using Genomatix MatInspector,^21^ we searched for putative p53REs in the promoters of the genes relative to MVA pathway. These genomic regions consisted of 3-kb upstream and 2-kb downstream relative to transcription start sites, whereas for FDFT1, MatInspector software identified three sites located into intron in gene-coding sequence. Each gene contained a putative p53RE in its promoter (Supplementary Table 2). In order to confirm that p53 binding to these sites, we performed chromatin immunoprecipitation (ChIP) in U343 cells, U251 cells transfected with an empty vector (mock) or a vector encoding the wild-type p53 and in NHA cells, treated or not with etoposide (Eto) at 25 *μ*M for 24 h. Then, we analyzed the amount of DNA precipitated with an antibody against p53 or a control antibody (IgG), using specific primers for the putative binding site located in promoters in different areas relative to transcriptional start site of each gene (Supplementary Table 3). Genomic regions of HMGCR, MVK, FDPS and FDFT1 were strongly enriched in the p53-immunoprecipitated chromatin, indicating that p53 is recruited to the consensus sequence of these genes (Figures 3a and b), whereas those of RabGGTA and LDLR were only enriched in the NHAs (Figures 3c and d). We validated the ChIP findings by analyzing the p53-binding sites at the canonical p53 target CDKN1A (p21waf). In order to distinguish between the endogenous and transfected p53, we performed a CHIP assay in U251 cells transfected with FLAG-p53 expression vector or with an equivalent amount of empty vector, as shown in Figure 3f. Then, we analyzed the amount of DNA immunoprecipitated with anti-FLAG antibodies or a control antibody (IgG), using specific primers for the putative binding site located in promoters in different areas relative to the transcriptional start site of each gene (Supplementary Table 3). Genomic regions of HMGCR, MVK, FDPS and FDFT1 were strongly enriched in the anti-FLAG-immunoprecipitated chromatin, indicating that the transfected p53 effectively is recruited to the consensus sequence of these genes (Figures 3e and g). Next, we tested the functionality of the identified p53REs. To this end, we cloned the DNA regions containing the putative p53RE of HMGCR MVK, FDPS, FDFT1, RabGGTA and LDLR into a luciferase reporter plasmid (Scheme 2; Supplementary Table 2) and carried out a series of promoter activity in U343 cells transfected with luciferase reporter plasmids bearing promoter and intronic regions of enzymes alone (Figure 4a) or in co-transfection with mutant p53 (R273H) (Figure 4b) and in U251 cells co-transfected with wild-type p53 compared with cells co-transfected with mock (Figure 4c) or reporter plasmids alone (Figure 4d). Luciferase expression was significantly enhanced in a p53-dependent manner in the constructs harboring the promoters of HMGCR, MVK, FDPS, FDFT1 (Figures 4a and d), whereas no luciferase activity in cell lines transfected with plasmids bearing RabGGTA and LDLR promoters was observed (data not shown). Moreover, in U343 cells, the same promoters showed reduced luciferase activity when co-transfected with the mutant p53 (Figure 4b). In order to confirm that the p53REs in the promoters are responsible for the elevation in luciferase activity, we mutated the genomic regions in the p53-binding sites. We carried out two types of mutations: (1) deletion of the region bearing the p53REs (Figure 4; Supplementary Table 4) and (2) single mutations of core nucleotides in the p53REs within the cloned fragment (Scheme 2; Supplementary Table 4). Very interestingly, all the mutants significantly reduced the p53-dependent induction of luciferase activity (Figures 4a and c). Taken together, these findings suggest that p53 could bind to the specific region of promoters of MVA metabolism-related genes inducing their transcription.

### p53 is recruited to the promoters of MVA pathway enzymes independently from SREBPs proteins

**Figure 5:**
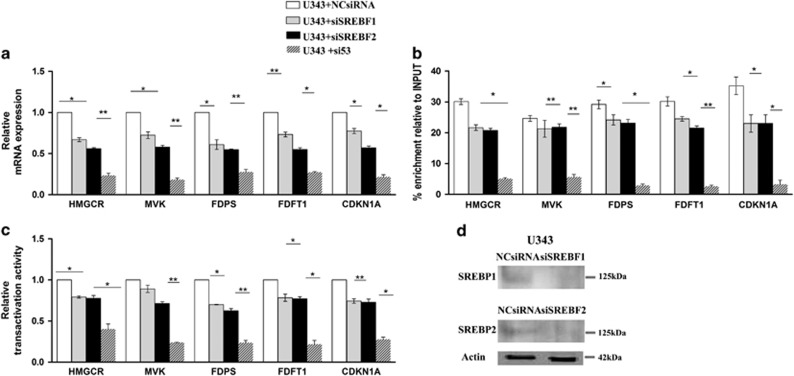
(**a**) qRT-PCR of enzymes related to MVA pathway, (**b**) ChIP assays and (**c**) luciferase reporter analysis of U343 cells after knockdown of SREBP1, SREBP2 and p53. U343 cells were treated with a control siRNA or siRNA directed against SREBP1, SREBP2 and p53. (**d**) Immunoblot shows knockdown of SREBP1 and SREBP2 (see also Figure 3f for the depletion of p53). Actin was made as control of loading. Data represent mean±S.D. of three independent experiments and statistical analysis was performed with one-way ANOVA, *P≤0.05; **P≤0.01

**Figure 6:**
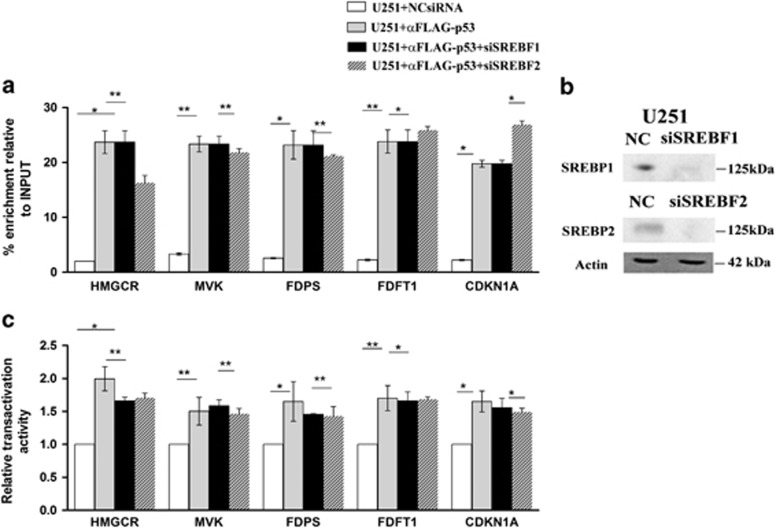
(**a**) ChIP assays and (**c**) Luciferase reporter analysis of U251 cells after knockdown of SREBP1 and SREBP2. U251 cells were co-transfected with FLAG-p53 and control siRNA or siRNA directed against SREBP1 and SREBP2. (**b**) Immunoblot shows knockdown of SREBP1 and SREBP2. Actin was made as control of loading. Data represent mean±S.D. of three independent experiments and statistical analysis was performed with one-way ANOVA, *P≤0.05; **P≤0.01

The physiologic regulation of MVA pathway enzymes requires the sterol regulatory element-binding proteins. Recently, Freed-Pastor *et al.*^22^ have described that the mutant p53 is recruited at the promoters of sterol genes by SREBPs to upregulate the expression of the sterol biosynthesis enzymes in human breast cancer cells. In order to test the physiological relevance of p53 into the transcriptional regulation of the enzymes of MVA pathway irrespective of SREBP proteins, we conducted qRT-PCR measurements of mRNA levels of aforementioned genes, experiments of ChIP and luciferase assays in U343 cells transfected with siRNA targeting SREBP1 and SREBP2. qRT-PCR measurements after depletion of SREBP1 and SREBP2 in U343 cells (as shown in Figure 5d) substantially decreased the expression levels of the enzymes but knockdown of p53 profoundly attenuated the enzymes expression (Figure 5a). Moreover, we carried out a quantitative ChIP analysis and observed that the ChiP signal was consistently reduced upon depletion of p53 in comparison with depletion of SREBP1 and SREBP2 (Figure 5b). Next, the activity of promoters of the enzymes revealed that the depletion of SREBP1 and SREBP2 slightly reduced the activation of a luciferase reporter under the control of cloned promoters of the enzymes in comparison with the depletion of p53 (Figure 5c). Next, to evaluate the effects of knockdown of SREBP1 and SREBP2 on the transcription of tested enzymes, we transiently co-expressed FLAG-p53 with specific siRNA of SREBP1 and SREBP2 in U251 cells (as shown in Figure 6b) and carried out a ChiP assay (Figure 6a). The chromatin region was immunoprecipitated with anti-FLAG, thus indicating the p53 binding to promoter regions of enzymes after depletion of SREBP1 and SREBP2 (Figure 6a). Luciferase assay of the enzymes revealed that the knockdown of SREBP1 and SREBP2 causes a weak and irrelevant reduction of the activity of promoter regions of enzymes (Figure 6c).

### p53 modulates CHO biosynthesis in glioblastoma cells

**Figure 7:**
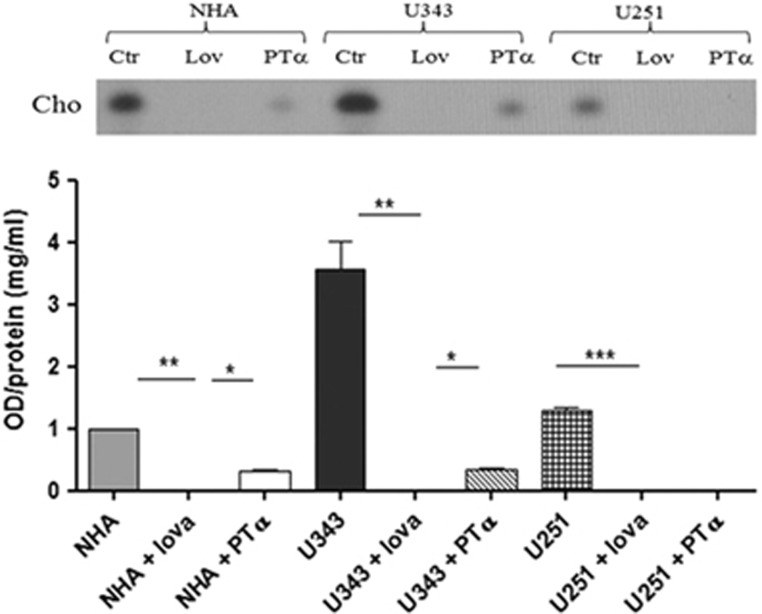
[^14^C]-acetate incorporation in neo-synthesized CHO. Chromatogram of non-saponifiable lipids from cells radiolabeled during a 14-h period following treatment. U343 and U251 cells were incubated with Lov (10 *μ*M for 24 h) and cyclic PT-*α* (30 *μ*M for 24 h). CHO bands are indicated with an arrow and the graphic represents densitometric analysis of two independent experiments. Statistical analysis was performed with one-way ANOVA *P≤0.05; **P≤0.01; ***P≤0.001

As p53 regulates the expression of some enzymes of MVA pathway, including HMGCR, the key regulatory enzyme of isoprenoids synthesis, we measured the synthesis of CHO product of MVA pathway in the human glioblastoma multiforme U343 and U251 cell lines and in NHA. In basal condition, we observed an increase in the incorporation of [^14^C]-acetate in CHO in U343 cells compared with U251 cells and their normal astrocytic counterpart (Figure 7). In any case, the presence of Lovastatin (Lov; 10 *μ*M for 24 h), a competitive inhibitor of HMGCR activity, inhibited the CHO synthesis in U343, NHA and U251cell lines; moreover, a transcriptional inhibitor of p53, cyclic pifithrin-alpha (PT-*α*; 30 *μ*M for 24 h) decreased the incorporation of [^14^C]-acetate in CHO product in all cell lines (Figure 7).

## Discussion

Malignant gliomas are highly dependent on the MVA pathway for the synthesis of CHO and isoprenoid compounds compared with their normal counterpart.^23^ This pathway is highly regulated in untransformed cells, and loss of any one of these regulatory mechanisms may contribute to dysregulation of this critical pathway and ultimately drive tumorigenesis.^24^ In this study, we analyzed the expression profiles of some enzymes relative to this pathway in two different cell lines of glioblastoma multiforme, U343 cells expressing a wild-type p53 and U251 cells containing a mutant p53 (R273H) allele lacking of transcriptional activity.^17^ We observed that HMGCR, MVK, FDPS, FDFT1, RabGGTA and LDLR are upregulated in U343 cells and in NHAs compared with U251 cells bearing mutant p53. We showed evidence that the expression of these genes is positively regulated by p53, indeed the depletion of p53 or the ectopic expression of mutant p53 in U343 cells reduces the levels of the examined enzymes, whereas the mRNA of CDKN1A, a known p53 target gene, is upregulated. Probably in this case, the regulation is p53 independent but others transcriptional factors^25^ may be involved. Taken together, these findings show that p53 binds to a well-defined p53-responsive element in the promoter region of HMGCR, MVK, FDPS and FDFT1, and is able to induce their transcription. Interestingly, p53 binds to the promoter regions of the RabGGTA and LDLR in NHAs but not in U343 cells. In the brain, the astrocytes are identified as producer of CHO secreted by lipoproteins and supply CHO to the neurons via LDLR.^26^ These results suggest the hypothesis that the transformation of these cells from normal to malignant is accompanied by a loss of p53 control of LDLR and RabGGTA, the latter being already described as a p53 target gene in human lymphoblastoid cell lines.^27^ How p53 discriminates among the several binding sites is unclear, although recent observations suggest that it may be due to aberrant DNA methylation and associated to chromatin changes.^28^ In this context, we observed that low levels of p53 in human glioblastoma cells can enhance the mRNA levels of HMGCR and of the other MVA pathway enzymes causing an increase of the metabolic flux through the MVA pathway contributing to the cell survival, according to the pro-survival roles of wild-type p53.^29^ Recent work by Freed-Pastor *et al.*^22^ showed that mutant p53 is recruited to genes that encode MVA pathway enzymes by SREBPs proteins to upregulate their expression in human breast cancer cells, contributing to the maintenance of the malignant phenotype. Here, we revealed that p53 binds to specific regions into the promoters of the genes relative to MVA pathway independently from SREBP1 and SREBP2 proteins. Indeed, ChIP analysis of p53 on promoter of enzymes after knockdown of SREBP1 and SREBP2 in U251 cell line with mutant p53 (R273H), revealed that p53 is recruited at the promoters in any case and with the same efficiency. Moreover, U251 cells express high level of SREBP1 and SREBP2, nevertheless they are unable to upregulate the sterol biosynthesis genes. These results suggest that p53 by itself can regulate the transcription of MVA pathway enzymes, however, we observed that in U343 cells the depletion of SREBP1 and SREBP2 slightly reduced the binding of p53 to the promoters suggesting that the transcriptional regulation by p53 may occur through several transcriptional co-regulators that have an integral role in the physiological functions of p53. The upregulation of MVA pathway in U343 cells caused a greater elevation in the *de novo* synthesis of CHO compared with U251 cells and astrocytes. In U251 cells, we observed a 40% decrease in total cell CHO. Furthermore, the treatment with cyclic PT-*α*, an inhibitor of the transcriptional activity of p53, inhibited the [^14^C]-acetate incorporation in CHO in U343 cells suggesting a correlation between p53 and CHO pathway. The results shown here suggest that in U343 cells, p53 could be the major regulator of the MVA pathway allowing the elevation of CHO metabolism essential for cellular survival. The hypothesis currently under investigation is that the upregulation of CHO metabolism could then drive tumorigenesis. Recent evidence have described that, although p53 is a tumor suppressor, some of its downstream targets do not have a tumor-suppressive function. The research of the pro-survival function of p53 is essential for cancer therapy ^30^ because the identification of novel treatments targeting the pro-survival function of p53 could direct the cancer cells toward apoptosis, thus allowing the acquisition of optimal efficacy for chemotherapy. We observed that U251 cells bearing mutant p53, although unable to upregulate the sterol biosynthesis genes, showed a high rate of endogenous CHO synthesis allowing the elevated rate of cell proliferation, to understand this mechanisms are needed for further studies. We believe that the reduction of sterol biosynthesis genes levels and, as consequence, a reduced amount of intracellular CHO make this cell type particularly sensitive to the inhibition of this pathway.^31^ Indeed, the treatment with Lov reduced considerably the amount of free CHO (Figure 7). In addition, most of glioma cells with wild-type p53 exhibit a high resistance to cytotoxic treatments used in clinical practice compared with glioma cells with transcriptionally inactive mutant p53.^32^ It appears that p53 activities associated with DNA repair contribute to the overall survival potential and drug resistance. Supporting the notion, there is evidence that chemoresistance mediated by p53 in glioma relies at least in part on the ability of p53 to activate the transcription of DNA repair genes,^33^ and of genes whose products directly contribute to drug resistance of glioma to temozolomide such as MGMT (O6-methylguanine-DNA methyltransferase), an enzyme that removes methyl and chloroethyl groups from the O6-position of guanine.^34^ Furthermore, the effects of p53 on drug resistance and the survival potential seen in different types of cancer cells, could be due to the activation of CHO pathway that, in order to support cell survival, may modulate resistance to a variety of anticancer drugs, within a functional phenotype termed multidrug resistance (MDR). The plasma membranes of MDR+ tumor cells are rich in fact, in CHO that assists the activity of Pglycoprotein (Pgp), an integral membrane transporter chemotherapy drugs. Another hallmark of MDR+ tumor cells is the increased prenylation and activity of GTP-binding proteins, which are also dependent on the rate of the MVA pathway activity.^35^ In summary, our results show that p53 is an essential transcription factor for the regulation of MVA pathway, and further studies will be necessary to determine the mechanism by which p53 regulates the MVA pathway enzymes. A better understanding of how p53 coordinates metabolic adaptation will facilitate the identification of novel therapeutic targets and will also illuminate the wider role of p53 in human biology.

## Materials and Methods

### Cell cultures and reagents

NHAs are normal human cells derived from human brain tissue and were cultured in AGM BulletKit medium (Lonza, Portsmouth, NH, USA), these cells were used as normal control system. Glioblastoma multiforme cell lines U343 and U251 cells (CLS, Berlin, Germany) were maintained in EMEM, supplemented with 10% fetal bovine serum (FBS), 2 mM l-glutamine, 0.1 mM non-essential amino acids, 1.0 mM sodium-pyruvate. Human embryonic kidney 293 cells (HEK293) were maintained in DMEM supplemented with 10% FBS. All cell lines were cultured at 37 °C in 5% CO_2_ controlled atmosphere. Reagents Eto, PT-*α* and Nut were purchased from Sigma-Aldrich S.r.l. (Milan, Italy). Cells were treated with Eto 25 *μ*M for 24 h, PT-*α* was used at a concentration of 30 *μ*M for 24 h, whereas Nut was administered at 10 *μ*M for 24 h. Lov, a gift from Dr. AW Alberts of the Merck, Sharp and Dohme Institute (Kenilworth, NJ, USA), was used at 10 *μ*M for 24 h of treatment. siRNA targeting SREBP1 (h) (sc36557) was purchased from Santa Cruz Biotechnology (Dallas, TX, USA). siRNA p53 (106141), SREBP2 (s27) and Lipofectamine RNAiMAX reagent were from Invitrogen (Carlsbad, CA, USA).

### Immunoblotting

Cells were harvested after 24-h treatment, washed twice with phosphate-buffered saline (PBS) and resuspended in lysis buffer with protease inhibitors (Hepes 50 mM, NaCl 150 mM, EDTA 50 mM, NaF 100 mM, Na ortovanadate 2 mM, glycerol, Na4P2O7 10 mM, 10% Triton pH 7.5, all compounds were from Sigma, Milan, Italy). Protein concentration was determined by Bio-Rad Protein Assay (Bio-Rad, Berkeley, CA, USA), 50 *μ*g of proteins were resuspended in Laemmli sample buffer and loaded onto a SDS–PAGE. Samples were resolved under constant voltage (40 mA) and transferred onto nitrocellulose blot (100 V). Blots were blocked in PBS containing 0.1% Tween 20 and 5% no fat dry milk for 1 h at room temperature. Filters were probed overnight with primary specific antibodies at 4 °C: mouse anti-p53 (sc-126), rabbit anti-p21waf (sc-756), rabbit anti-HMGR (sc-271595), rabbit anti-MVK (sc-366285), rabbit anti-FDFT1 (sc-99145), rabbit anti-RabGGTA (sc-133617), mouse anti-LDL (sc-18823), anti-mouse SREBP1(sc365513) and anti-SREBP2 (sc-5603) were from Santa Cruz Biotechnology, rabbit anti-FDPS (ab-38854), anti-FLAG was from Sigma. Immunodetection of specific proteins was carried out with horseradish peroxidase-conjugated goat anti-mouse or anti-rabbit IgG (Bio-Rad) for 1 h at room temperature and visualized using the Enhanced Chemiluminescence (ECL) system (Amersham, Little Chalfont, Buckinghamshire, UK), images were scanned with Bio-Rad's Chemidoc system.

### Quantitative real-time PCR

Total RNA was isolated using the NucleoSpin RNA II kit (Macherey-Nagel, Mountain View, CA, USA). Complementary DNA (cDNA) was transcribed using SuperScript II Reverse Transcriptase (Invitrogen), starting from 1 *μ*g of high pure RNA. MVA genes expression profiles were evaluated with specific primer sets (Supplementary Table 1) and using SsoFast EvaGreen reagents (Bio-Rad), b2-microglobulin was used as housekeeping gene. qRT-PCR protocol was: a pre-heating step for 3 min at 95 °C, 40 cycles at 95 °C for 10 s and 60° for 30 s and last end-step at 65 °C for 10 s. Results were analyzed with 2^-ddCt^ method.^36^

### Plasmid constructs

Human p53 expression plasmids pCMV-p53wt and pCMV-p53 R273H plasmid was purchased by Addgene (#16439, Cambridge, MA, USA),^37^ pcDNA3 flag p53FLAGp53 was purchased by Addgene (plasmid #10838).^38^ MVA genes' promoter constructs containing p53REs were amplified with the high fidelity Taq AccuPrime Pfx DNA Polymerase (Invitrogen). Protocol was: 2 min at 95 °C followed by 28 cycles 15 s at 95 °C, 30 s at 55–58 °C and 1 min at 70 °C using primer sets showed in Supplementary Table 2 and 4. Products were digested as *Kpn*I/*Hind*III fragments and ligated into the pGL3-Basic Vector firefly-luciferase reporter-gene vector (Promega, Madison, WI, USA). Restriction enzymes and the T4 DNA ligase were from New England BioLabs (Ipswich, MA, USA). Promoter constructs with deletion of specific p53REs were assembled using same cloning protocol and using primer sets described in Supplementary Table 4. Promoter constructs carrying mutations in the p53REs were obtained by site-directed mutagenesis kit, QuikChange II-E Site-Directed Mutagenesis Kit (from Agilent Technologies, Santa Clara, CA, USA) and mutagenic primers (Supplementary Table 4). All constructs confirmed to be the sequence of interest by sequencing. Cell transfection-U251 cells at 90% of confluence were transfected with 5 *μ*g of pCMVp53 plasmid using Lipofectamine 2000 (DNA/Lipofectamine ratio was 1/2.5) and were harvested 48 h after transfection. U343 cells were transfected with 5 *μ*g of mut(R273H)p53 plasmid, whereas HEK293 cells were transfected with 5 *μ*g of pCMVp53 or co-transfected with a total amount of 6 *μ*g DNA (pCMVp53+mut(R273H)p53 plasmids). Six hours after transfection, incubation medium was replaced with fresh and FBS supplemented medium. Control cells were transfected with empty vector (mock).

### Chromatine immunoprecipitation

ChIP experiments were carried out with MAGnify Chromatin Immunoprecipitation System (Invitrogen) using a minimum of 6x106 cells. U251 cells at 90% of confluence were transfected with 2 *μ*g of pCMVp53 plasmid using Lipofectamine 2000 and were harvested 48 h after transfection. Cells were harvested and washed with PBS, then chromatin/proteins complexes were cross-linked with 1% of formaldehyde for 10 min, then cells were lysed and DNA were sonicated with BRANSON digital sonifier 251 (Medford, NY, USA), to have DNA fragments of 500–1000 base pairs. An aliquot of sheared chromatin was stored and processed as DNA input control, whereas 300 *μ*l of sonicated chromatin solution were incubated overnight at 4 °C with 5 *μ*l of rabbit anti-human p53 polyclonal antibody (Life Technology, Carlsbad, CA, USA). As negative control (non-IP), a chromatin fraction was incubated in parallel with rabbit IgG. All antibodies were coupled to magnetic beads (Dynabeads, Waltham, MA, USA). Finally, DNA was de-cross-linked and purified using DNA Purification Magnetic Beads. Isolated DNA was subjected to qRT-PCR analysis using specific primes (Supplementary Table 3), results are shown as chromatin enrichment to input and are adjusted for their respective non-IP.

### Reporter gene assays

Cells were plated in 48-well plates in triplicates (5 × 10^4^ cells per well) and 24 h later were transfected using Lipofectamine 2000 (Invitrogen) with 1 *μ*g per well of pGL3 plasmids harboring the promoters of the mentioned genes (Supplementary Table 2), and or 1 *μ*g per well of pCMV-p53wt a or empty vector (mock). To normalize transfection efficiency for reporter vector assays, cells were co-transfected with b-galactosidase (b-Gal) plasmid (500 ng per well) (Promega). Forty-eight hours after transfection, cells were harvested and washed with PBS and then lysed with the reporter lysis buffer (Promega). Luciferase activities in cell lysates were measured with the Luciferase System Assay (Promega) and read at luminometer and normalized to control b-Gal activity.

### [^14^C]- acetate uptake

Actively growing, subconfluent cells were incubated with Lov (10 *μ*M for 24 h) and PT-*α* (30 *μ*M for 24 h). Afterward, cells were incubated with 20 *μ*l of 2-14C-acetate([^14^C]- acetate) (Specific activity: 59mCi/mM from Perkin Elmer, Waltham, MA, USA) for 12 h. Cells were washed and harvested with cold PBS, resuspended in 500 *μ*l of isopropanol and sonicated. After centrifugation, supernatants were dried and dissolved in 50 *μ*l of chloroform, whereas pellets were solubilized in 500 *μ*l di 0.1 M NaOH for protein assay. In all, 10 *μ*l of each sample were loaded on Thin Layer Cromatography – Silica gel-60G (Millipore, Billerica, MA, USA) in presence of 1,2-^3^H(N)cholesterol standard (Specific activity 60.0 Ci/mM from NEN products – DuPont, Boston, MA, USA) and developed in hexane/diethylether/acetic acid (70 : 30 : 1) used as solvent system.^39^ Membranes were exposed to X-ray film and film developed, spots were quantified by densitometry using a digital imaging system (Image Lab software, Berkeley, CA, USA).

### Statistical analysis

Data are reported as mean±S.D. of three independent experiments. When comparing two means, statistical significance (*P*≤0.05) was assessed by Student's two-tailed *t*-test. When comparing more than two experimental groups, we used one-way ANOVA statistical analysis (*P≤0.05; **P≤0.01; ***P≤0.001).

## Figures and Tables

**Figure:**
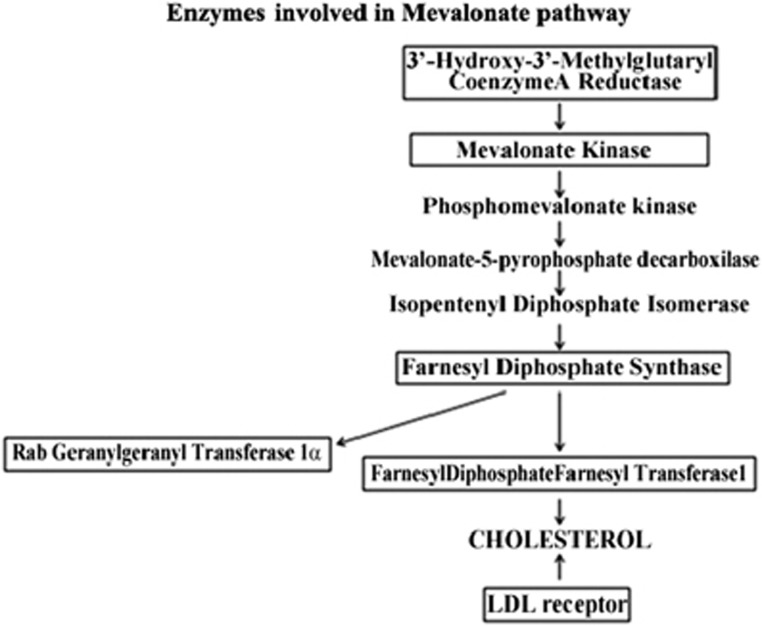
Scheme 1 Enzymes involved in MVA pathway

**Figure:**
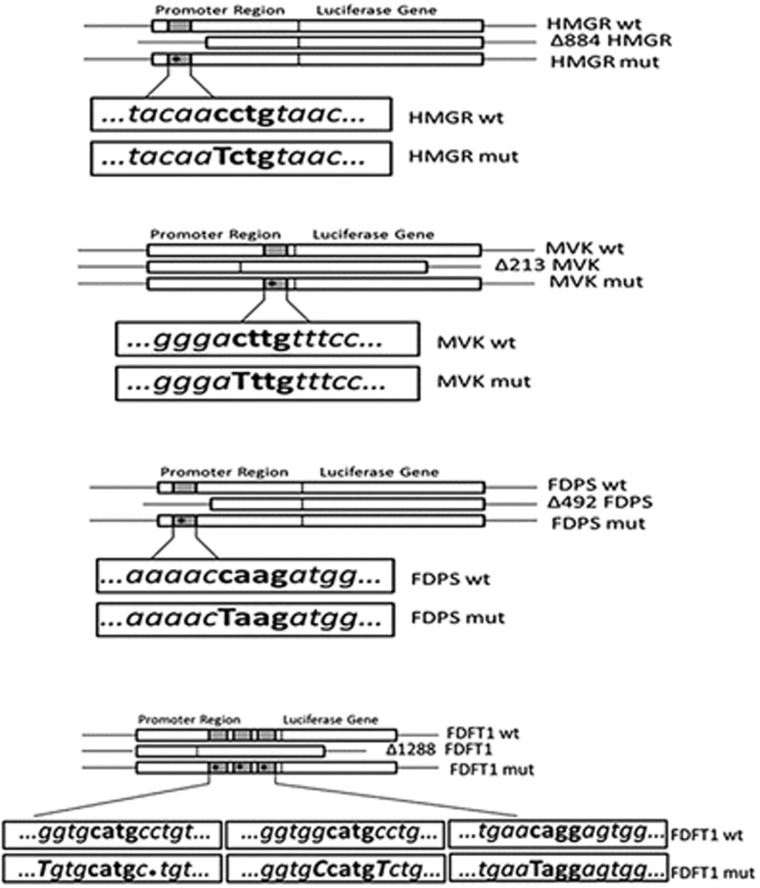
Scheme 2 Promoter regions containing p53REs cloned in pGL3 luciferase reporter vector. p53REs positive to ChIP assays were cloned and integrated in a pGL3 luciferase reporter vector to evaluate the link between these potential promoter regions and p53 transcriptional activity. Regions cloned from HMGCR, MVK, FDPS, FDFT1 genes were also deleted and mutated in p53RE and subsequently tested for luciferase transcription ability

## Abbreviations

MVA: mevalonate
HMGCR: 3′-hydroxy-3′-methylglutaryl-coenzyme A reductase
MVK: mevalonate kinase
FDPS: farnesyl diphosphate synthase
FDFT1: farnesyl diphosphate farnesyl transferase 1
RabGGTA: Rab geranylgeranyl transferase 1*α*
LDLR: LDL receptor
Eto: etoposide
PT-*α*: pifithrin-alpha
Nut: nutlin-3a
Lov: lovastatin
CHO: cholesterol
