# Supplementary material for: p53 regulates the mevalonate pathway in human glioblastoma multiforme
Source: Cell Death Dis. 2015 Oct 15;6(10):e1909–. doi: 10.1038/cddis.2015.279 (PMC4632304; doi:10.1038/cddis.2015.279)

**p53 regulates the Mevalonate pathway in human glioblastoma multiforme**

Alba D’Alessandro^1,2^*, Chiara Laezza^3^*, Luciano Di Croce^4^, Paola Picardi^1,2^, Elena Ciaglia^1,2^, Simona Pisanti ^1,2^, Anna Maria Malfitano^1,2^, Marika Comegna^5,6^, Raffaella Faraonio^5,6^ , Patrizia Gazzerro ^1,2^, Maurizio Bifulco^1,2^.

**Authors’ Affiliations**: 1Department of Medicine and Surgery, University of Salerno, 84081 Baronissi (SA); 2Department of Pharmacy, University of Salerno, Via Giovanni Paolo II, 132, 84084 Fisciano Salerno (SA), Italy; 3Institute of Endocrinology and Experimental Oncology, IEOS CNR, 80131 Naples, Italy; 4Centre de Regulacio Genomica (CRG), Universitat Pompeu Fabra, Dr.Aiguader 88, 08003 Barcelona, Spain; 5Department Molecular Medicine and Medical Biotechnologies, University of Naples "Federico II", via S. Pansini,5, 80131 Naples Italy; 6CEINGE, Biotecnologie Avanzate, Naples, Italy

Figure S1 HMGR, MVK, FDPS, FDFT1, RabGGTA and LDLR expression in different cell lines. (a) qRT-PCR analyses in adenocarcinoma breast cancer cell line MCF and MDA MB 231 cell lines. (b) ) qRT-PCR analyses in colon cancer cell lines HCT116 and colorectal adenocarcinoma SW620. Data represent mean ± SD of three independent experiments


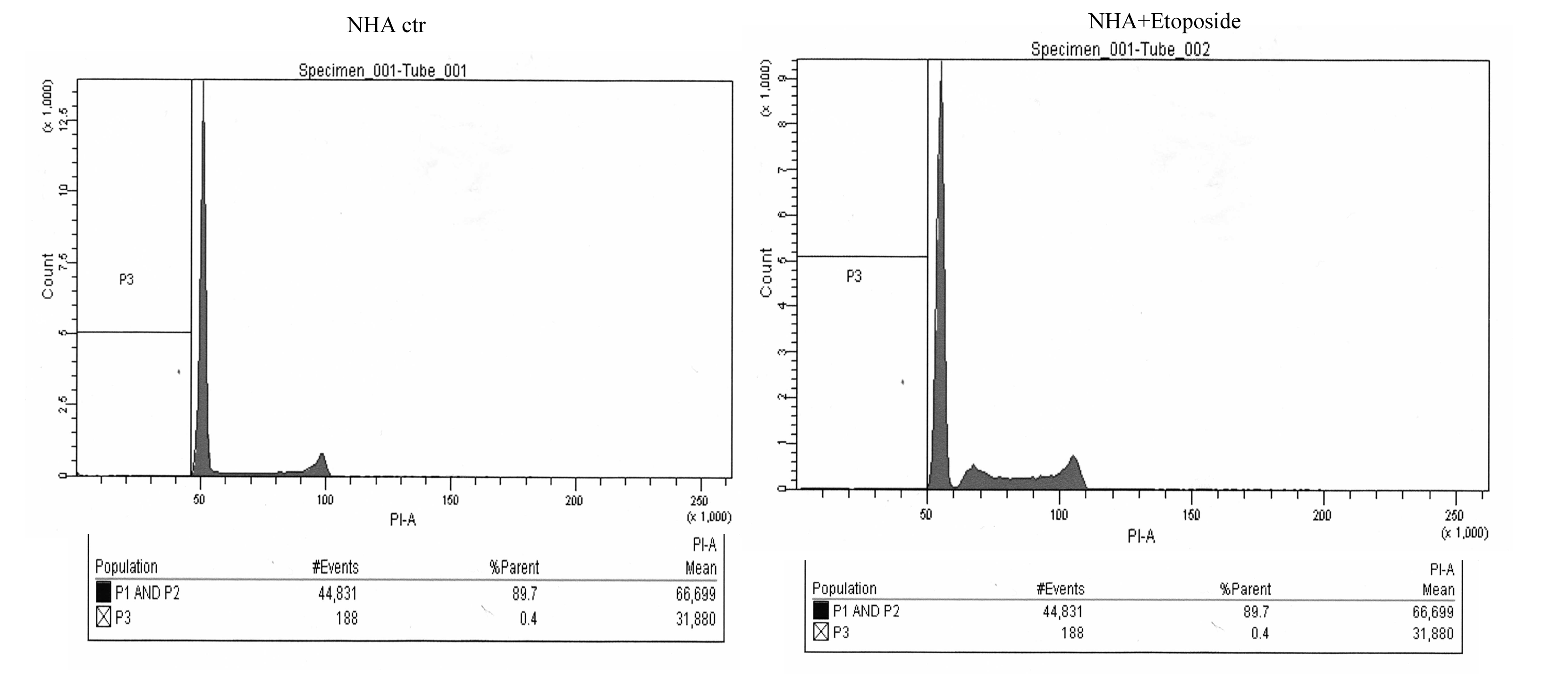


**Figure S2** Cell cycle analysis. NHA untreated and treated with 25 mM of etoposide

Su**pplementary table**

**Table 1. qRT-PCR primer sets**. All primers were optimized at Tm 60°C. β2microglobulin (β2M) was the housekeeping gene


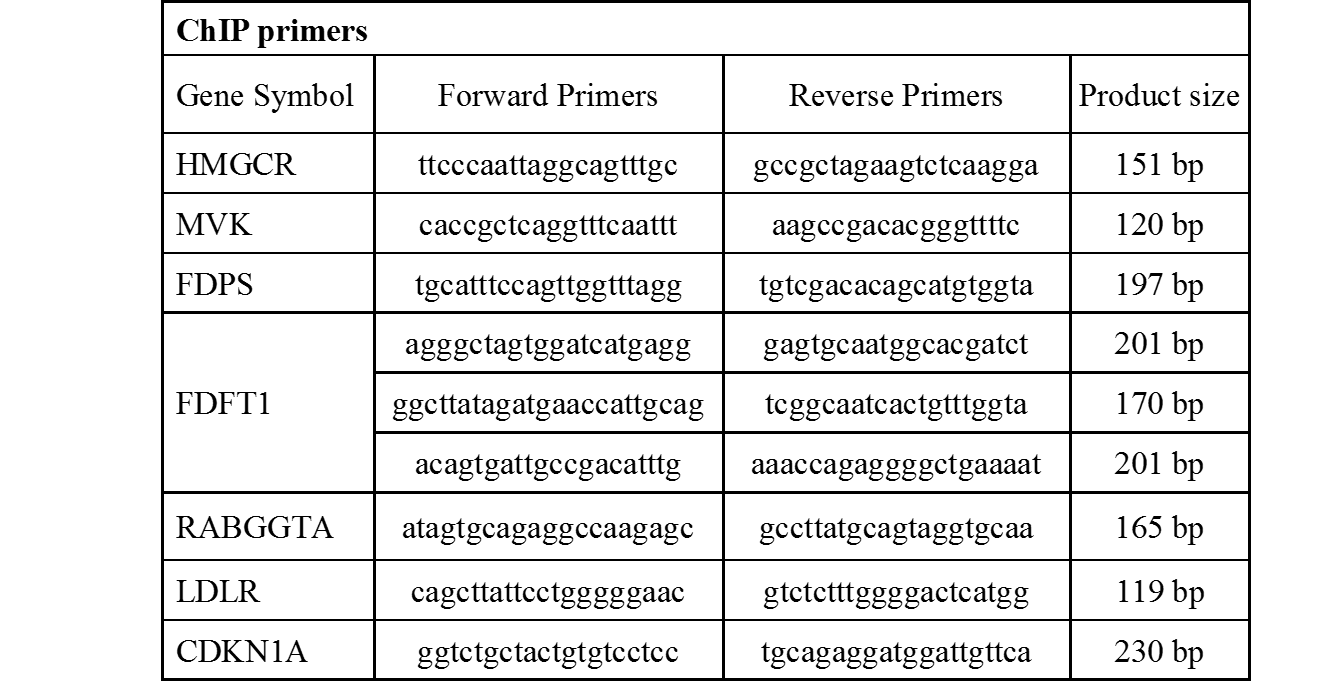


**Table 2. ChIP primer sets**. All primers were optimized at Tm 60°C.


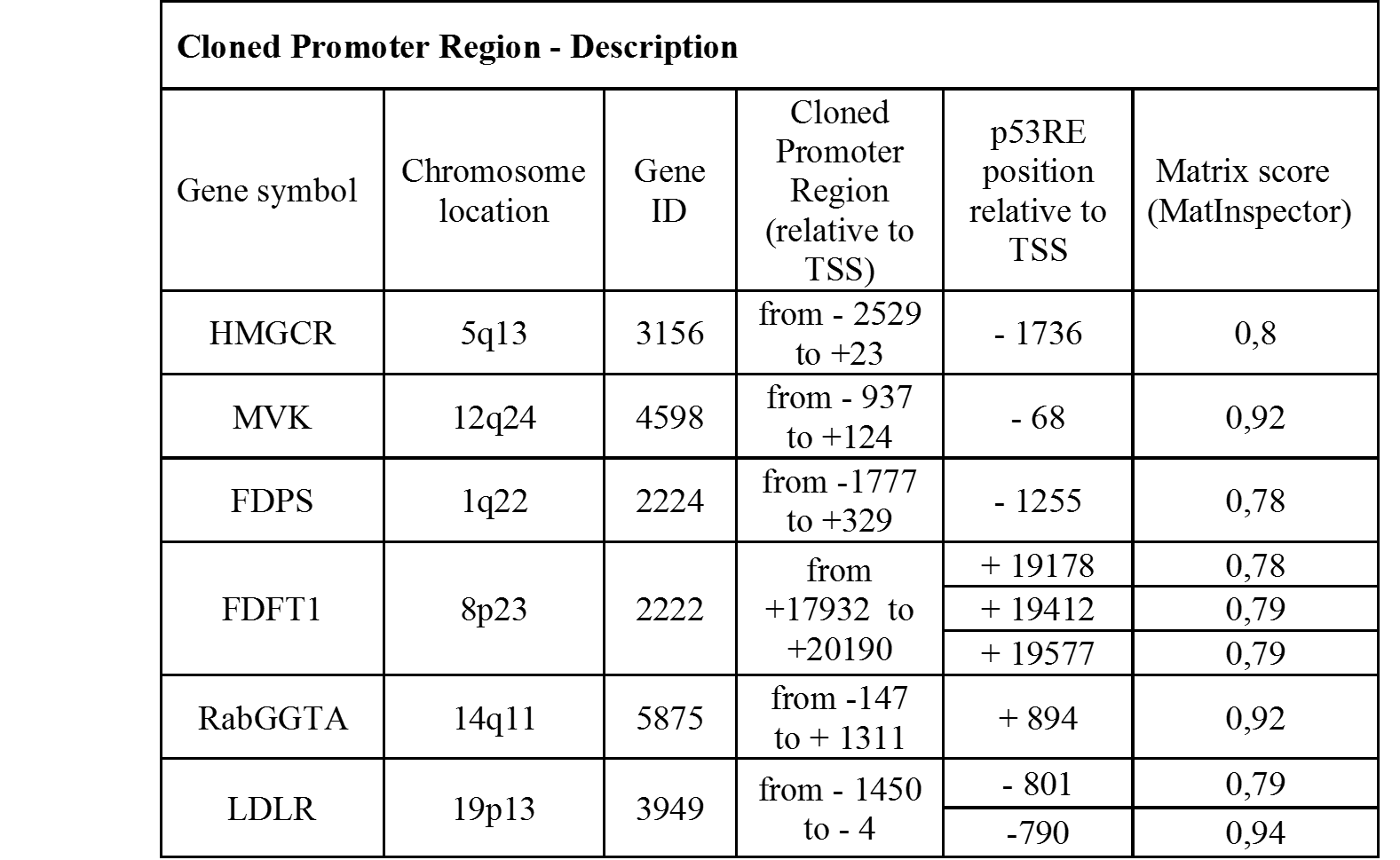


**Table 3. Cloned promoter regions - Description.** Description of p53 Responsive Elements containing regions with position relative to Transcription Statr Site (TSS) and MatInspector’s putative site score.

**Table 4. Cloned Constructs – Primer sets.** Specific primer sets were used to cloned p53REs containing sequences and to induce mutations in cloned constructs. Primers used to clone p53REs regions hold a 5’- tail recognised by restriction enzymes, respectively: -GGTACC is target of KpnI endonuclease and -AAGCTT is recognized by HindIII enzyme. Primers used to induce deletions used same principles, while primers cointaining mutations were used to induce mutations in plsmids containing constructs using the QuikChange II- XL Site-Directed Mutagenesis kit


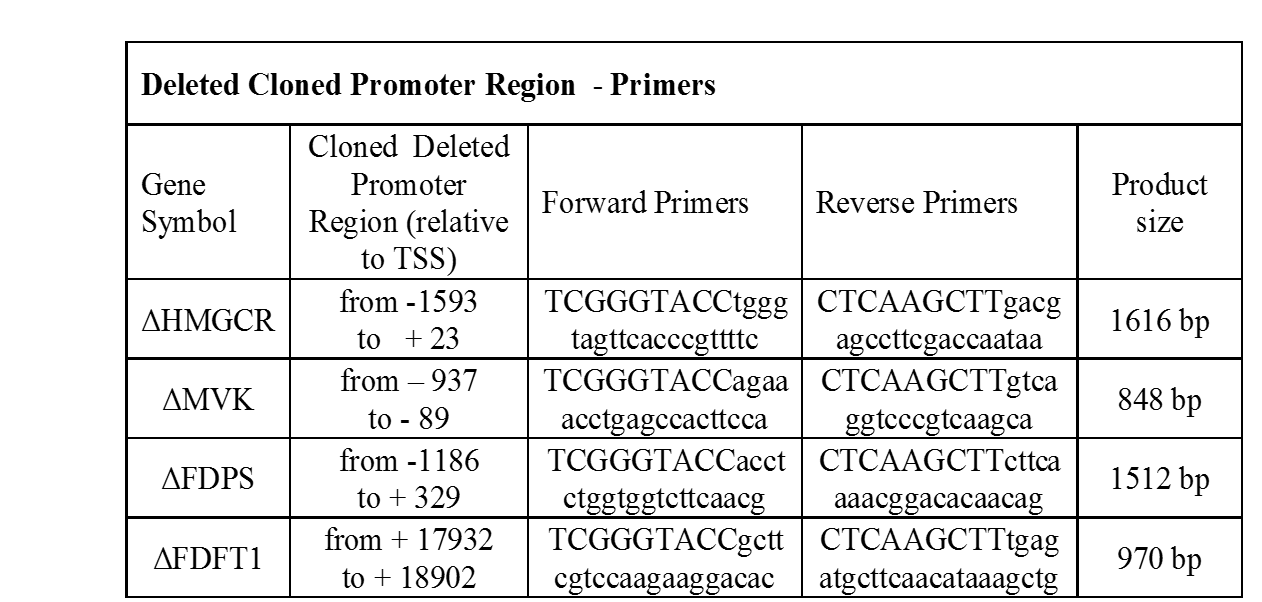

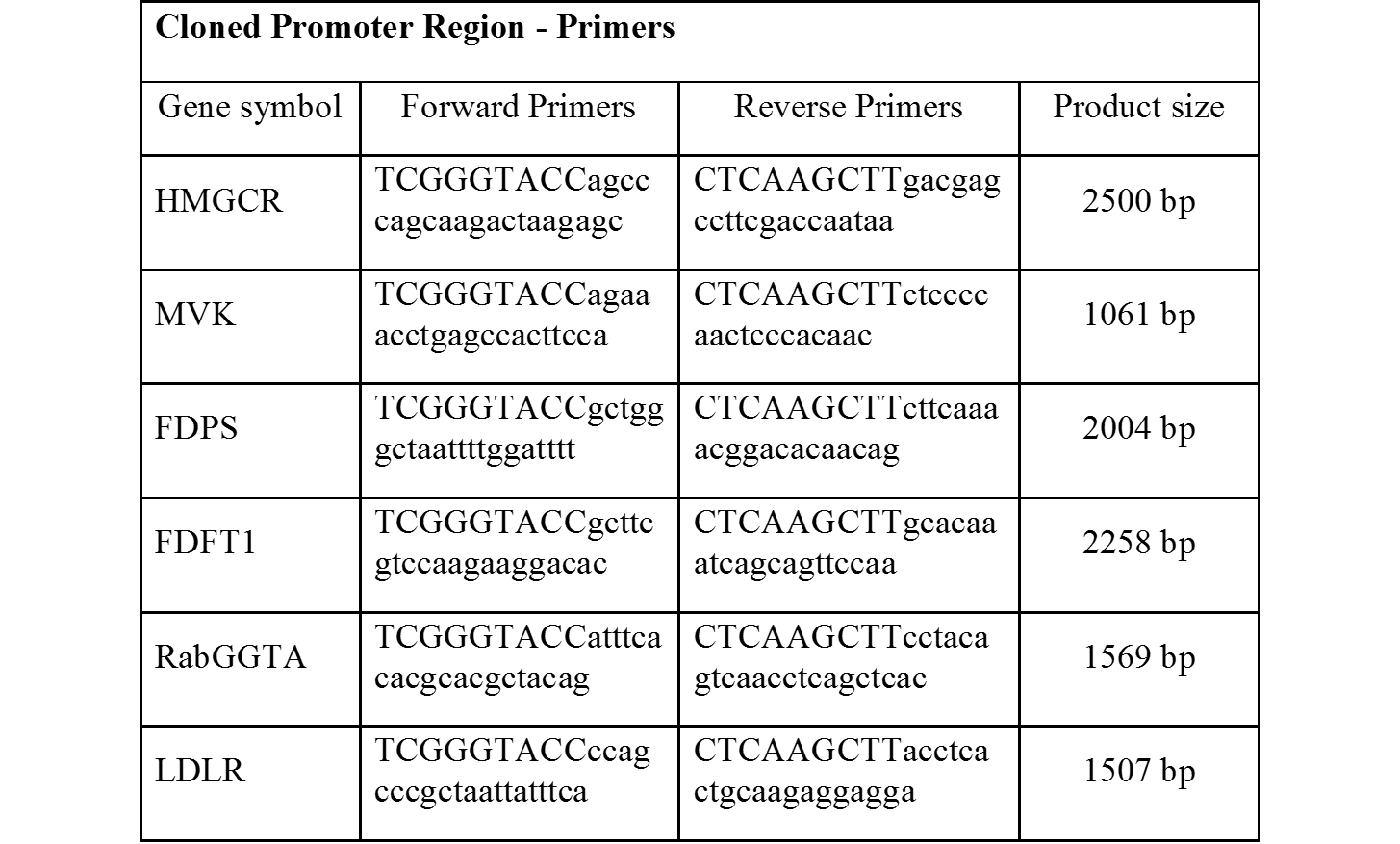

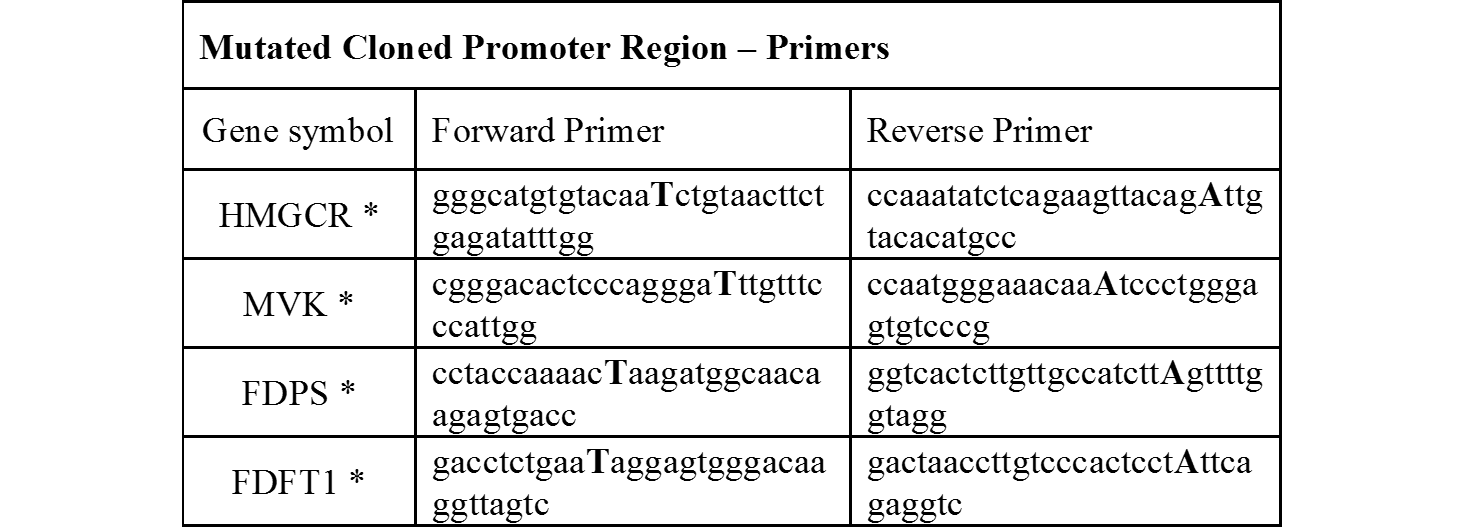

Supplement: Supplementary Information [file cddis2015279x1.doc]
